# Association of Motoric Cognitive Risk Syndrome and High C-Reactive Protein Serum Levels With Incident Major Neurocognitive Disorder: Results From the Quebec NuAge Cohort

**DOI:** 10.1093/gerona/glae260

**Published:** 2025-02-06

**Authors:** Olivier Beauchet, Kevin Galéry, Pierrette Gaudreau, Gilles Allali

**Affiliations:** Departments of Medicine and Geriatrics, University of Montreal, Montreal, Quebec, Canada; Research Centre of the Geriatric University Institute of Montreal, Montreal, Quebec, Canada; Research Centre of the Geriatric University Institute of Montreal, Montreal, Quebec, Canada; Department of Medicine, University of Montreal, Montreal, Quebec, Canada; Research Centre of Centre Hospitalier de l’Université de Montreal, Montreal, Quebec, Canada; Leenaards Memory Center, Department of Clinical Neurosciences, Lausanne University Hospital and University of Lausanne, Lausanne, Switzerland; (Biological Sciences Section)

**Keywords:** C-reactive protein, Gait, Observational cohort, Risk factor

## Abstract

Both motoric cognitive risk (MCR) syndrome and C-reactive protein (CRP) serum levels have been separately associated with increased risk of incident major neurocognitive disorder. The study aims to compare the CRP serum levels of older adults with and without MCR and to examine the associations of MCR and CRP serum levels and their combination with incident major neurocognitive disorder.

915 individuals participating in an older adult’s population-based observational cohort study with a 3-year follow-up design were selected. MCR and CRP serum levels were collected at baseline. Incident major neurocognitive disorder was measured at annual follow-up visits using the Modified Mini-Mental State Examination (≤79/100) and simplified instrumental activity daily living scale (<4/4) score values.

The prevalence of MCR at baseline assessment was 3.7%. The overall incidence of major neurocognitive disorder was 3.0%. MCR alone (hazard ratio = 25.36 with 95% confidence interval = [6.25–102.95] and *p* ≤ .001) and MCR with a high CRP serum level (hazard ratio = 5.61, with 95% confidence interval [1.29–24.26] and *p* = .021) were significantly associated with incident major neurocognitive disorder.

MCR is a significant risk factor for predicting major neurocognitive disorder in older adults, while serum CRP levels are not. In addition, serum CRP levels reduce the predictive strength of MCR for major neurocognitive disorder.

Cognitive complaints associated with slow walking speed define motoric cognitive risk syndrome (MCR) (1). This clinical syndrome is associated with an increased risk of incident major neurocognitive disorder, which refers to the new onset of a significant decline in one or more cognitive domains that interferes with independence in daily activities, regardless of the etiology (2,3). The pathogenesis of MCR is still discussed, but it has been suggested that inflammation may be an important contributing factor (4). Inflammation is a biological mechanism postulated to play a role in both the decline in cognitive and physical performances of older adults (5). C-reactive protein (CRP) is predominantly produced by the liver in response to inflammatory signals. Its blood levels increase in response to acute and chronic inflammation, making CRP a commonly used marker to assess the inflammation status in clinical and research settings (6). For instance, a high CRP serum level has been associated with an increased risk of incident major neurocognitive disorder, including Alzheimer and non-Alzheimer diseases (7,8). Several potential mechanisms related to inflammation may explain this association. First, chronic inflammation can lead to the disruption of the blood–brain barrier, allowing inflammatory molecules and cells to enter the brain, which can contribute to neural damages and consecutive cognitive decline (9). Second, elevated CRP is a marker of systemic inflammation, which can trigger microglial activation in the brain (10). Activated microglia release pro-inflammatory cytokines that can damage neurons and synapses, contributing to cognitive decline. Third, in Alzheimer’s disease, chronic inflammation is thought to exacerbate the deposition of amyloid-beta plaques. CRP and other inflammatory markers can enhance amyloid-beta production and aggregation, leading to further neural damage (11). Fourth, inflammation can also influence tau pathology, which is another hallmark of Alzheimer’s disease. Elevated CRP and other inflammatory cytokines can promote tau hyperphosphorylation and aggregation, contributing to neurofibrillary tangles and neuronal death (11,12). Fifth, inflammation is a risk factor for cardiovascular diseases, which in turn can contribute to vascular dementia (13). Elevated CRP levels are associated with atherosclerosis and other vascular conditions that can impair cerebral blood flow, leading to cognitive decline (13). Sixth, inflammatory processes can increase oxidative stress, which damages cellular components, including DNA, proteins, and lipids. Oxidative stress in neurons can impair their function and survival, leading to cognitive deficits (14).

Higher CRP serum levels have been reported in individuals with MCR compared with those without MCR (5). However, the effect of an association of MCR with CRP serum levels on the risk of major neurocognitive disorder in older adults has not yet been reported. As both MCR and high CRP serum levels have been separately associated with incident major neurocognitive disorder, we hypothesized that their association could increase the risk of incident major neurocognitive disorder in older adults. We examined this hypothesis in the Quebec Longitudinal Study on Nutrition and Successful Aging (NuAge Study), an observational cohort study of aging participants recruited in the province of Quebec (Canada) (15). In this cohort study, clinical information was collected through validated questionnaires from which it could be determined the annual incidence of major neurocognitive disorders, including Alzheimer and non-Alzheimer dementia, merged in one simple group. The NuAge participants were recruited between 2003 and 2005, when the reported annual incidence of major neurocognitive disorders was between 10% and 20% in community-dwelling older adults (16). The aim of the present study was to compare the CRP serum levels of NuAge participants with and without MCR and to examine the associations of MCR and CRP serum levels and their combination with incident major neurocognitive disorder.

## Method

### Design and Population

The NuAge Study is a longitudinal observational cohort study that followed older community dwellers annually for 3 years after recruitment (15). Men and women aged 67–84 years, without cognitive impairment (ie, Modified Mini-Mental State Examination (3MS) score >79/100) and physical disability (ie, able to walk 300 m and to climb one storey without rest) and living independently, were randomly recruited between November 2003 and June 2005 (15,17). Among the 1 753 participants recruited at baseline and who agreed to the inclusion of their data into NuAge database and biobank for future studies, we excluded 66 (3.8%) participants who used a walking aid (an exclusion criterion for computing MCR status), 507 (28.9%) who had missing clinical and biological information, 227 (12.9%) who were lost over the 3-year follow-up period, and 39 (2.2%) who withdrew consent to the use of their data. The remaining subset was composed of 915 (52.2%) participants.

### Assessment

Age, sex, Caucasian status, and number of medications taken daily were recorded at baseline. The presence of cardiovascular risk factors (coded yes vs no) were noted and included current smoking status; being overweight and obese (if a body mass index of ≥25 kg/m^2^ was present); a low level of physical activity defined as a score below the lowest tertile (ie, <69.1 for female and <87.7 for male) of the physical activity scale score for the older adults; hypertension defined as noted positive on the Older Americans Resources and Services Multidimensional Functional Assessment (OARS) questionnaire, or if the participant was taking antihypertensive medications, or had a blood pressure measurement >140/90 mmHg; and diabetes considered present when it was self-reported, or noted positive on the OARS questionnaire or if the participant was taking antidiabetic medication or had a fasting glycemia of >6.9 mmol/L (18,19). Cardiovascular diseases (ie, heart, limb, and cerebrovascular disease) and musculoskeletal diseases recorded with the OARS questionnaire were also noted (13). Depression was assessed with the 30-item geriatric depression scale (GDS) and considered present when its score was >10/30 (20). Walking speed was measured over a 4-m distance at usual pace twice. After the initial baseline assessment, the full clinical assessment was repeated annually over a 3-year follow-up period, and related data were collected.

Finally, fasting serum levels of CRP were measured at baseline from −80 °C frozen samples by CDL Laboratories Inc., Montreal, CA, using a high-sensitivity particle-enhanced immuno-turbidimetric assay (Roche Diagnostics Canada, Laval, CA). Analyses were performed on a Cobas 8000/module 702 (Roche Diagnostics). The intra- and inter-assay coefficients of variation were 0.7%–0.8% and 1.3%–1.5%, respectively. The CRP serum level mean value ± standard deviation (*SD*) and its highest tertile, defined as serum concentration >2.85 mg/L, were used as outcomes.

### Definition of Motoric Cognitive Risk Syndrome

Subjective cognitive complaint was defined as a “yes” response to the question, “*Do you feel you have more problems with memory than most others*?” of the 30-item GDS (20). Slow walking speed was considered if walking speed was at least 1 *SD* below the age and sex-appropriate mean values established in the present cohort. The combination of these 2 components was used to define MCR as described by Verghese et al. (1) in participants without major neurocognitive disorders and walking disability at baseline.

### Definition of Major Neurocognitive Disorder

The 3MS score defined the cognitive performance at baseline (T1) and at each annual subsequent visit (T2, T3, and T4) (7). From T2 to T4, incident major neurocognitive disorder was considered present if the 3MS score was ≤79/100 and the simplified instrumental activity daily living score was ≤4/4 (17,21).

### Standard Protocol Approval and Patient Consents

The Research Ethics Boards (REB) of the University Institutes of Geriatrics of Sherbrooke and the “*Institut universitaire de gériatrie de Montréal*” approved the NuAge Study protocol. The NuAge Database and Biobank have been approved by the REB of the CIUSSS de l’Estrie-CHUS (Québec, Canada). Participants provided written informed consent, and from the initial cohort of 1 793 participants, 1 753 (98%) agreed to the inclusion of their data and biological samples into the NuAge Database and Biobank for future studies. The REB of the Jewish General Hospital (Montreal, Quebec, Canada) approved the study.

### Statistics

Means, *SD*, and percentages described the participants’ characteristics. The highest tertile of circulating CRP level was defined as a serum concentration >2.85 mg/L. Participants were separated into 2 groups based on their MCR status. Group comparisons were performed using unpaired *t*-tests, Mann–Whitney tests, or Chi-squared tests. A multiple logistic regression was performed to examine the association of MCR (used as an independent variable) with the highest tertile of CRP serum level (used as the dependent variable) adjusted for the participants’ baseline characteristics. A multiple Cox regression was also performed to examine the association of MCR, the highest tertile of CRP serum level, and their combination (used as independent variables in the same model using non-MCR with a normal CRP serum level as the reference group) with overall incident major neurocognitive disorder over the 3-year period of follow-up (used as the dependent variable) adjusted for the participants’ baseline characteristics. In addition, the reliability of 3MS and instrumental activities of daily living (IADL) scores was examined using the Cronbach’s alpha coefficient. The results were 0.886 and 0.777, respectively, showing that there was a good internal consistency for 3MS and an acceptable internal consistency for IADL. *p*-Values <.05 were considered statistically significant. All statistics were performed using SPSS (version 29.0; SPSS, Inc., Chicago, IL).

## Results

The prevalence of MCR at baseline assessment was 3.7%. The overall incidence of major neurocognitive disorders was 3.0%. Participants with MCR were less frequently Caucasian (*p* = .003) and had a higher incidence of major neurocognitive disorder (*p* ≤ .001) compared with those without MCR (Table 1). The mean value of CRP serum levels (*p* ≤ .001) and the prevalence of its highest tertile (*p* ≤ .001) were higher in MCR participants compared with those without MCR. The multiple logistic regression showed that there was, at baseline, no significant association between MCR and the highest tertile of CRP (odds ratio [OR] = 1.17 with 95% confidence interval [CI] = [0.54–2.53] and *p* = .684). The multiple Cox regression showed that MCR alone (hazard ratio [HR] = 25.36 with 95% CI = [6.25–102.95] and *p* ≤ .001) and MCR with the highest tertile of CRP (HR = 5.61, with 95% CI[1.29–24.36] and *p* = .021) were significantly associated with incident major neurocognitive disorders (Table 2).

**Table 1.** Baseline Characteristics of Participants According to Their Motoric Cognitive Risk Syndrome Status (*n* = 915)

	Motoric Cognitive Risk Syndrome		*p*-Value*
	Yes (*n* = 34)	No (*n* = 881)	
Age (year), mean ± *SD*	75.4 ± 4.1	73.8 ± 4.1	.855
Female, *n* (%)	15 (44.1)	453 (51.4)	.403
Caucasian, *n* (%)	32 (94.1)	874 (99.2)	**.003**
Medication taken daily, *n* (%)			
Mean ± *SD* (*n*)	5.9 ± 3.3	4.6 ± 3.1	.997
Polypharmacy†, *n* (%)	21 (61.8)	403 (45.7)	.066
Cardiovascular risk factors‡, *n* (%)	33 (97.1)	792 (89.9)	.169
Cardiovascular diseases§, *n* (%)	15 (44.1)	314 (35.6)	.312
Musculoskeletal disorders‖, *n* (%)	22 (64.7)	529 (60.0)	.586
Depression¶, *n* (%)	4 (11.7)	100 (11.4)	.625
Incident major neurocognitive disorder#, *n* (%)	7 (20.6)	20 (2.3)	≤**.001**
C-reactive protein serum level			
Mean ± *SD* (mg/L)	6.0 ± 12.1	3.1 ± 4.3	≤**.001**
Highest tertile**, *n* (%)	24 (70.6)	0	≤**.001**

*Comparison based on unpaired *t*-test, Mann–Whitney or Chi-square test, as appropriate.

†Number of therapeutic drugs taken daily ≥5.

‡Possessing a minimum of one of the following risk factors: smoking, overweight status or obesity, low level of physical activity defined as a score below the lowest tertile (ie, <69.1 for female and <87.7 for male) of the physical activity scale for the older adults, hypertension or diabetes.

§Possessing a minimum of one of the following diseases: heart disease, peripheral vascular disease or cerebra vascular disease.

‖Based on the Older Americans Resources and Services Multidimensional Functional Assessment (OARS) questionnaire information, 3 dichotomous (ie, yes vs no) categories of diseases were created to indicate the presence of cardiovascular disease (associating heart, peripheral, and cerebrovascular disease), musculoskeletal disorders (including tendinitis, osteoarthritis, arthritis, and fibromyalgia), and other diseases.

¶30-item geriatric depression score >10/30.

#Cognitive performance evaluated at baseline (T1) and at each annual subsequent visit (T2, T3 and T4) using the 3MS score (7). From T2 to T4, incident dementia was considered present if the 3MS score was ≤79/100 and the IADL score was ≤6/8.

**Defined as a serum concentration >2.85 mg/L. *P*-value significant in bold (i.e, below 0.004 because of multiple comparisons *n* = 12).

**Table 2.** Multiple Cox Regressions Showing the Association of Motoric Cognitive Risk Syndrome, the Highest Tertile of C-Reactive Protein Serum Level and Their Combination (Used as Independent Variables in the Same Model Using the Participants Without Motoric Cognitive Risk Syndrome With a Normal C-Reactive Protein Serum Level as the Reference Group) With Overall Incident Major Neurocognitive Disorder Over the 3-Year Period of Follow-up (Used as the Dependent Variable) Adjusted by the Participants’ Baseline Characteristics (*n* = 915)

	HR	[95% CI]	*p*-Value
Ref*	–		
MCR alone	25.36	[6.25–102.95]	≤**.001**
C-reactive protein alone	1.82	[0.65–5.04]	.253
MCR and C-reactive protein	5.61	[1.29–24.26]	**.021**

*Notes*: CI = confidence interval; HR = hazard ratio; MCR = motoric cognitive risk syndrome.

*Participants without motoric cognitive risk syndrome and with a C-reactive protein level below the highest tertile, defined as a serum concentration >2.85 mg/L; Cox model adjusted by age, sex female, Caucasian, polypharmacy, depression, cardiovascular risk factor, cardiovascular diseases and musculoskeletal disorders.

## Discussion

The present findings revealed mixed results. First, the MCR NuAge participants had a significantly higher CRP serum level at baseline compared with their counterparts free of MCR, although no significant association between MCR and a high CRP serum level was found at baseline. Second, we found that whether or not the combination of MCR with a high CRP serum level was significantly associated with incident major neurocognitive disorder, this risk was lower compared with the risk of MCR alone. In addition, there was no significant association between a high CRP serum level and an incident major neurocognitive disorder.

NuAge participants with an MCR status had a higher baseline CRP serum level compared with those without MCR, but the multiple logistic regression revealed that there was no significant association between MCR and a high CRP serum level. These mixed results are in accordance with a previous study performed in a community-dwelling Chinese population of individuals aged 60 and over (5). In this study, the mean value of CRP serum level was significantly higher in MCR participants compared with non-MCR participants, but the highest tertile of CRP was not associated with MCR status (OR = 1.21 with 95% CI = [0.89–1.63] with *p* = .219) (4). This absence of association between CRP used as a continuous value and MCR status was also reported in a meta-analysis including 5 aging cohorts (4). One explanation of this result may be that MCR and CRP serum levels are both associated with the same chronic age-related morbidities, such as cardiovascular diseases, diabetes, obesity, and arthritis (22). The prevalence of these chronic morbidities was high in the NuAge participants, which exposed participants to a combination of both MCR and CRP serum levels without any causal relationship. Indeed, when diseases are common, more people are likely to have multiple of them. By chance alone, there will be more participants who have both diseases compared with when one or both diseases are rare. This overlap can create a statistical association even if the diseases do not directly influence each other.

The results showed that MCR alone and MCR with a high CRP serum level were associated with an increased risk of incident major neurocognitive disorder. This risk was 7 times greater for MCR alone than for the combination of MCR with a high CRP serum level. Like previous studies, our results confirmed that MCR is a pre-dementia stage because it is strongly associated with an increased risk of incident major neurocognitive disorder (1,2). However, to the best of our knowledge, the significant association between a combination of MCR with a high CRP serum level and incident major neurocognitive disorder is being reported for the first time. Three complementary explanations of this association may be suggested. First, inflammation is increasingly recognized as a significant factor in the pathogenesis of major neurocognitive disorders (7,8). High circulating levels of inflammatory markers like CRP are indicators of chronic inflammation, which is thought to contribute to the cascade of events leading to neuronal damage and cognitive decline (5–8). This inflammation can exacerbate the progression from MCR to major neurocognitive disorder by accelerating neuronal loss or dysfunction. Second, both MCR and elevated CRP serum levels are associated with vascular health risk factors (3,6,22). MCR can be linked to poorer vascular health due to its association with physical frailty and slower walking speed, while high CRP serum levels are a known risk factor for cardiovascular diseases (23–25). Poor vascular health can lead to cerebrovascular damage—a well-known risk factor for vascular dementia—and can also contribute to major neurocognitive disorders such as Alzheimer’s pathology (26–29). Third, an interaction effect may be suggested. The presence of MCR suggests existing cognitive impairment and possibly compromised brain functions, while a high CRP serum level indicates systemic inflammation. These conditions may interact in a way that the inflammation exacerbates the neurological effect of MCR, speeding up the transition to overt major neurocognitive disorder.

Our results also highlight that the combination of MCR and high CRP serum levels did not increase the risk of incident major neurocognitive disorders, as the risk was higher with MCR alone. In addition, a high CRP serum level was not associated with incident major neurocognitive disorders, whereas it was previously shown that there was a significant association (7,8). Altogether, these results reflect a complex interaction between MCR and the circulating CRP serum level for the risk of incident major neurocognitive disorder. Several reasons may be suggested. First, CRP is a general marker of inflammation and is elevated in a variety of conditions, including infections, autoimmune diseases, and cardiovascular diseases (6). This lack of specificity means that elevated CRP levels do not uniquely indicate neuroinflammation or neurodegenerative processes associated with dementia. Second, many older adults have elevated CRP levels due to other common age-related conditions, such as arthritis or cardiovascular disease (30). This can confound the relationship between CRP and major neurocognitive disorder, making it difficult to determine if elevated CRP is specifically related to cognitive impairment. Third, the predictive value of CRP for dementia may be influenced by the timing and duration of inflammation. Short-term elevations in CRP may not have the same implications as chronic, long-term inflammation. Most studies measure CRP at a single time-point, which may not accurately capture the inflammatory processes relevant to dementia development (31). Fourth, major neurocognitive disorder is influenced by a multitude of factors, including genetic, lifestyle, and other biological markers (7,8). These markers often provide more specific and actionable information for predicting dementia risk.

MCR is considered as predementia stage like minor neurocognitive disorder, suggesting that motor impairment could be the first biomarker of cognitive impairment (1–3). However, it may be suggested that neurocognitive disorders, especially in non-Alzheimer’s dementia, may cause motor impairment and thus MCR rather than merely a consequence. For instance, it has been reported that older adults with minor neurocognitive disorder had a higher risk of developing motor impairments and falls compared with those without minor neurocognitive disorder (32). This supports the idea that cognitive decline can precede and contribute to motor risks. In addition, it has been reported that executive dysfunction was strongly associated with gait abnormalities in older adults, indicating that cognitive impairments can directly affect motor control (33). The degeneration of brain areas involved in both cognitive and motor functions, combined with research findings that demonstrate a predictive relationship between cognitive decline and motor impairments, supports the causal role of neurocognitive disorders in MCR (34). This highlights the importance of early cognitive assessments in identifying individuals at risk for motor decline and consecutively implementing preventive strategies.

Our study benefits from a robust methodology, featuring a large sample size of NuAge participants and a 3-year period of prospective and observational follow-up, which are significant strengths. However, it has some limitations that warrant discussion. Notably, the incidence of major neurocognitive disorder observed in our study was relatively low at 3.0%, which is lower than that reported in prior studies (16). This reduced incidence indicates that the NuAge cohort primarily consisted of healthier older adults. Additionally, it raises the possibility that the incidence of major neurocognitive disorder may have been underestimated due to the diagnostic criteria employed. Typically, major neurocognitive disorder diagnoses are confirmed through an interdisciplinary evaluation process, utilizing comprehensive neuropsychological assessments and brain imaging, which provides a more thorough understanding of the condition. Moreover, the value of self-reported symptoms in our study may affect both MCR and major neurocognitive disorder diagnoses. This limitation is not specific to our study, but it is a general limitation related to both diagnoses. Self-reported questionnaires are associated with different biases, like the desirability or response bias, which may result in invalid answers (35). However, self-report assessment is a usual way to collect information in epidemiological studies. In addition, we used validated questionnaires to collect the items required for diagnoses of MCR and major neurocognitive disorder, and this collection of information was not only self-reported but collected by experienced health professionals. Finally, both MCR and major neurocognitive disorder diagnoses are dependent not on 1 criterion but on 2 criteria, which lead to several advantages, like increased accuracy. Using multiple criteria often helps to ensure that a diagnosis is more accurate. However, in some cases, this can also filter out people who are unnecessary if one of the diagnostic components is biased. Nevertheless, because self-reported items form 1 component in both the MCR and major neurocognitive disorderdiagnoses and, thus, if some participants endorse all self-reported symptoms, the findings from this study would be biased away from the null. Another limitation is related to the fact that we only have information on MCR and CRP at baseline assessment. It may be suggested that an alternative viable working theory is that heightened CRP causes increased risk of both MCR (at least in some individuals) and major neurocognitive disorder (most likely, among those with high MCR).

## Conclusion

This Quebec population aging study confirmed, firstly, that MCR is a risk factor that predicts major neurocognitive disorder in older adults. Secondly, CRP serum levels did not predict major neurocognitive disorder in older adults. Thirdly, the high CRP serum level diminished the strength of MCR risk predicting major neurocognitive disorder. Future studies should confirm this association in other populations, especially in a population with more age-related comorbidities and chronic inflammatory conditions.
